# Successful Implementation of Single Urine Polymerase Chain Reaction Test for Evaluating Suspected Cytomegalovirus Infection in Neonates

**DOI:** 10.1097/pq9.0000000000000586

**Published:** 2022-08-01

**Authors:** Shabih Manzar, Patricia Pichilingue-Reto, Ramachandra Bhat

**Affiliations:** From the * Section of Neonatology, Department of Pediatrics, Louisiana State University Health Sciences Center, Shreveport, LA; † Section of Pediatric Infectious Diseases, Department of Pediatrics, Louisiana State University Health Sciences Center, Shreveport, LA.

## Abstract

Overuse of laboratory investigations is viewed as medical waste. In the past, to diagnose congenital cytomegalovirus (CMV) infection, consecutive urine culture samples were obtained. With the advent of polymerase chain reaction (PCR) technology, 1 urine specimen should be enough. We conducted this quality improvement study to look at the effect of a practice change from 3 to 1 urine specimen for PCR testing.

**Methods:**

The authors instituted a single PCR urine test for CMV in their neonatal intensive care unit (NICU) in May 2021. We reviewed the data on all the urine CMV PCRs obtained on neonates for 1 year, May 1, 2020, to April 30, 2021 (Epoch 1), and compared it with the data obtained from May 1, 2021, to February 28, 2022 (Epoch 2).

**Results:**

A total of 3,612 neonates were born during the study period—1,816 infants were born during Epoch 1 and 1,796 infants during Epoch 2. A total of 97 neonates (5.3%) were evaluated for congenital CMV infection during Epoch 1 and 149 infants (8.2%) during Epoch 2. The single urine sample CMV evaluation rate during Epoch 1 was 53.6% (52 infants out of 97 infants evaluated), which increased to 98.6% in Epoch 2 (147 infants out of 149 infants), *P* < 0.001. The monthly average cost per infant declined from a mean value of 70.1 dollars to a mean value of 39.5 dollars.

**Conclusions:**

We increased the single specimen urine CMV PCR test from 53.6% to 98.6%. The intervention resulted in reducing waste and improving resource utilization.

## INTRODUCTION

Worldwide cytomegalovirus (CMV) is the most common congenital viral infection, with a birth prevalence of 0.6% to 0.7%.^1,2^ To differentiate congenital infection from early postnatal infection, viral isolation should be performed within the first 2 to 3 weeks of life.^3,4^ Virus isolation by culture from urine or saliva has long been the standard method for diagnosing congenital CMV infection.^4^ Detection of CMV can be accomplished through routine viral culture (RVC) methods or centrifugation-enhanced rapid shell vial cultures (SVC). The RVC turn-around times range from 1 to 2 weeks, but sometimes it can take up to 4 to 6 weeks, whereas the SVC methods require only 12 to 72 hours.^5-7^

Furthermore, many neonatologists and pediatricians ordered multiple cultures to ensure CMV detection in infected infants. In 1988, Demmler and colleagues^8^ identified 41 urine specimens positive by PCR assay from a total of 44 specimens positive by tissue culture. No positive PCR results were found in 27 urine specimens that were negative by tissue culture. This study showed that PCR amplification was a valuable tool for diagnosing congenital CMV infection.^8^ Further studies showed that real-time PCR was highly sensitive (100%). In contrast, the sensitivity of rapid culture was 89.3% when considering real-time PCR as a reference for CMV detection in neonatal urine.^9^ Consensus recommendations published in 2017 established that the diagnosis of congenital cytomegalovirus-infected neonates should include real-time PCR of saliva, urine, or both within the first 3 weeks of life.^10^ In our institution, in the transition from urine culture to PCR for CMV testing, the practice of obtaining 3 consecutive urine samples in line with the previous practice of obtaining 3 CMV cultures persisted, despite PCR being a different test with higher sensitivity. This often leads to infants’ discharge delays. Moreover, an unnecessary economic burden is the consequence of the persistence of the original practice of obtaining 3-urine samples. Hence, we intended to implement a practice change to ordering a single urine CMV PCR test for all suspected congenital CMV cases through a Quality Improvement (QI) initiative.

## METHODS

### Setting

The current study was a single-institution QI Study conducted at a Level 3B Neonatal Intensive Care Unit with a 40-bed capacity and Level-1 Newborn nursery, located at St. Mary Medical Center, Louisiana State University Health Sciences, Shreveport. The Institutional Review Board (IRB) determined that the proposed QI activity was not research involving human subjects as per regulations and waived the need for consent. The center supports approximately 2,000 deliveries per year, with an average of 600 NICU admissions per year. A diverse population of preterm infants, late preterm infants, clinically ill term neonates, and neonates with diverse medical and surgical conditions are being cared for at the NICU. In total, 8 neonatal nurse practitioners (NP), 5 neonatologists, and 3−4 resident physicians care for the neonates admitted to the NICU at this academic institution. Well-appearing neonates born at 35 weeks and older’ gestational age are admitted to the Newborn nursery under the pediatric hospital medicine service. A total of 3−4 hospitalist physicians, 1 NP, and 1−2 resident physicians provide care to the neonates in the nursery.

### Description of Problem

**Fig. 1.**
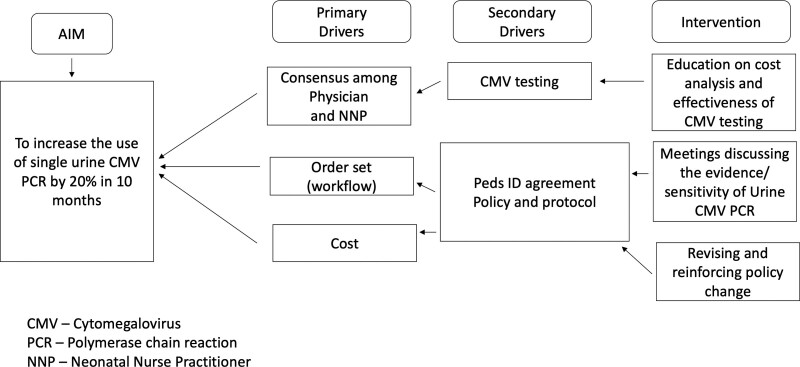
Key driver diagram.

At our institution, the urine CMV PCR test orders are entered by the residents and NPs. The indications for ordering urine CMV PCR were suspected congenital CMV infection, mostly neonates with documented intrauterine growth restriction (IUGR) without apparent reasons (maternal preeclampsia, placental insufficiency). Through the help of the laboratory supervisor, the data on all the urine CMV PCR tests obtained on neonates for the 1 year before the intervention, May 1, 2020, to April 30, 2021, were extracted. The data evaluation revealed a high prevalence rate of 3-urine samples per infant for congenital CMV evaluations using a molecular diagnostic assay with PCR technique. After evaluating the baseline data and identifying causes of evaluating suspected congenital CMV infection with PCR tests on multiple urine samples, we formulated a SMART AIM and key driver diagram of the project to guide the implementation of QI measures (Fig. 1). We aimed to increase the use of single urine sampling for CMV PCR testing by 20% in 10 months.

### Interventions

We formed a multidisciplinary team including neonatologists, pediatric infectious diseases physicians, hospitalists, NICU, and newborn nursery NP. The team was led by the primary investigator (PI, first author). Education was delivered to all clinicians through emails and face-to-face interaction. The program was discussed periodically in the division quality assurance meeting fortnightly (see Supplemental Digital Content, http://links.lww.com/PQ9/A397). Indications for congenital CMV infection evaluations, sensitivity data on urine CMV DNA PCR tests, and the cost involved for unnecessary 3 urinary samples were discussed. The knowledge gaps contributed to increased healthcare costs for the patients and a longer length of stay. Furthermore, a delay in the timely discharge of newborn infants due to a longer time needed for the procurement of 3-urine samples was also discussed. After reaching a consensus among all the stakeholders, the order set was updated. We implemented the practice change starting May 2021.

### Measures

Our study included 3 outcome measures: congenital CMV infection evaluation cost per infant, congenital CMV infection evaluation rate, and single urine sample CMV evaluation rate. For the current study, we defined CMV infection evaluation as a screening evaluation for congenital CMV infection utilizing urine CMV PCR tests. Congenital CMV infection evaluation cost per infant was calculated based on the laboratory charges per urine sample obtained per infant. The values were calculated every month for deriving time-series data points to monitor the process change over time. Congenital CMV infection evaluation rate was defined as the total number of infants with congenital CMV evaluations per 1,000 live births (LB). The congenital CMV evaluation rate was calculated for each month and monitored over time. Due to the ease of collection of urine samples using a single urine sample and the reinforcement of targeted neonatal CMV screening based on clinical symptomatology and risk factors of congenital CMV infection, we expected the monthly congenital CMV infections evaluation rate to increase. Hence, the latter was used as an outcome measure. The single urine sample CMV evaluation rate was calculated as the percentage of infants evaluated for congenital CMV infection through urinary DNA PCR testing with a single urine sample among all the infants evaluated for congenital CMV infections. The monthly single urine sample CMV evaluation rate, which is an outcome measure, was also used as a process measure for the current study as this quality metric directly impacted the cost per infant and congenital CMV evaluation rate and tracked the quality improvement process. The balancing measure for the current study was urine CMV DNA PCR positivity rate, defined as the proportion of urine PCR tests positive for CMV DNA among the neonates born during the study period.

### Data Source and Data Analysis

We used Epic as our electronic health records. Through the help of the laboratory supervisor, the data on all the urine CMV PCR tests obtained on neonates for the 1-year prior, May 1, 2020, to April 30, 2021, were extracted and entered onto an Excel sheet (Epoch 1). These data served as the baseline or pre-QI-phase data. Epoch 2 (QI-phase) was from May 1, 2021, to February 28, 2022, a prospective phase following the implementation of QI interventions that began on May 1, 2021. The monthly congenital CMV infection evaluation rate and congenital CMV infection initial evaluation cost per infant were plotted using the U-chart and X-bar-R Statistical Process Control (SPC) charts, respectively.^11^ The process measure, single urine sample evaluation rate, was depicted using a P-chart. Upper control limit (UCL) and lower control limit (LCL) for both the SPC charts were set at ±3 sigma. Special cause variations and centerline shifts were identified using the standard Montgomery rules.^12,13^ A special cause signal shift was indicated by the centerline shift and recalculation of UCL and LCL. We reanalyzed for stability after the centerline shift to detect any special cause variation. The conventional statistical tests were used to compare the single sample evaluation (chi-square test) and urine CMV PCR tests positivity rates (Fisher’s exact text) between the 2 epochs. QI Macros add-in software (KnowWare International Inc., Denver, Colorado; version 2022) was used to create SPC charts and to identify special cause variations.

## RESULTS

**Fig. 2.**
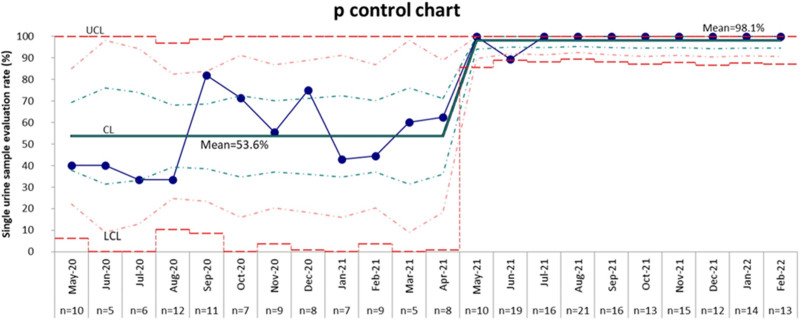
P-chart depicting monthly single urine sample CMV evaluation rate. X axis shows months and the total number of infants evaluated for congenital CMV (n) during each specific month. CMV, cytomegalovirus.

**Fig. 3.**
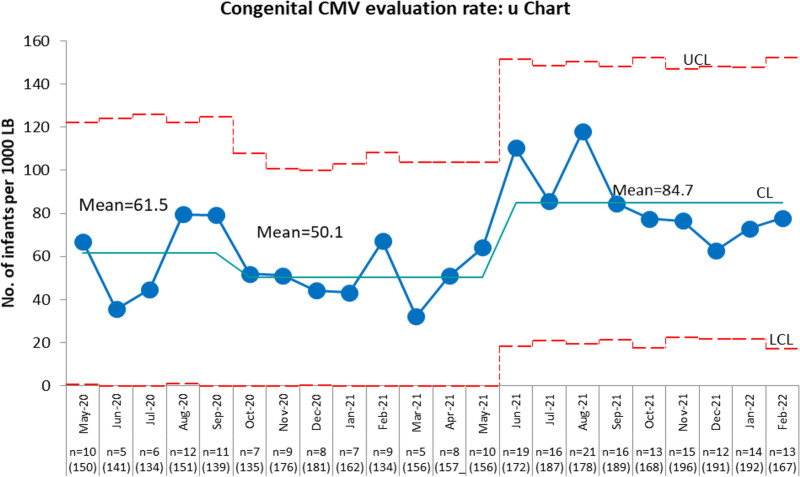
Statistical process control U-chart depicting the monthly congenital CMV evaluation rate expressed as the number of infants evaluated with urine CMV for congenital CMV infection per 1,000 Live Births (outcome measure). X axis indicates months, total no of infants evaluated for congenital CMV (n) and total no. LB during each month (in bracket). CMV, cytomegalovirus; LB, live births.

A total of 3,612 neonates were born during the study period among which 1,816 infants were born during Epoch 1 and 1,796 infants during Epoch 2. A total of 97 neonates (5.3%) were evaluated for congenital CMV infection during Epoch 1 and 149 infants (8.2%) during Epoch 2. The single urine sample CMV evaluation rate during Epoch 1 was 53.6% (52 infants out of 97 infants evaluated). Following the implementation of QI intervention, the single urine sample CMV evaluation rate increased significantly to 98.6% (147 infants out of 149 infants), *P* < 0.001. The P-chart (Fig. 2) depicts a special cause signal shift indicated by the centerline shift after the introduction of the QI intervention, which suggests that we achieved the intended goal (outcome measure) and the process of improvement was on the right track (process measure). During Epoch 1, the pre-QI baseline phase, the initial congenital CMV evaluation rate was 61.5 per 1,000 LB and it decreased to a new baseline rate of 50.1 per 1,000 LB. A month after the implementation of QI intervention, a valid process change in the congenital CMV evaluations rate occurred, leading to an increase in the rate of congenital CMV evaluations from a baseline rate of 50.1 per 1,000 LB to a new rate of 84.7 per 1,000 LB (Fig. 3).

**Fig. 4.**
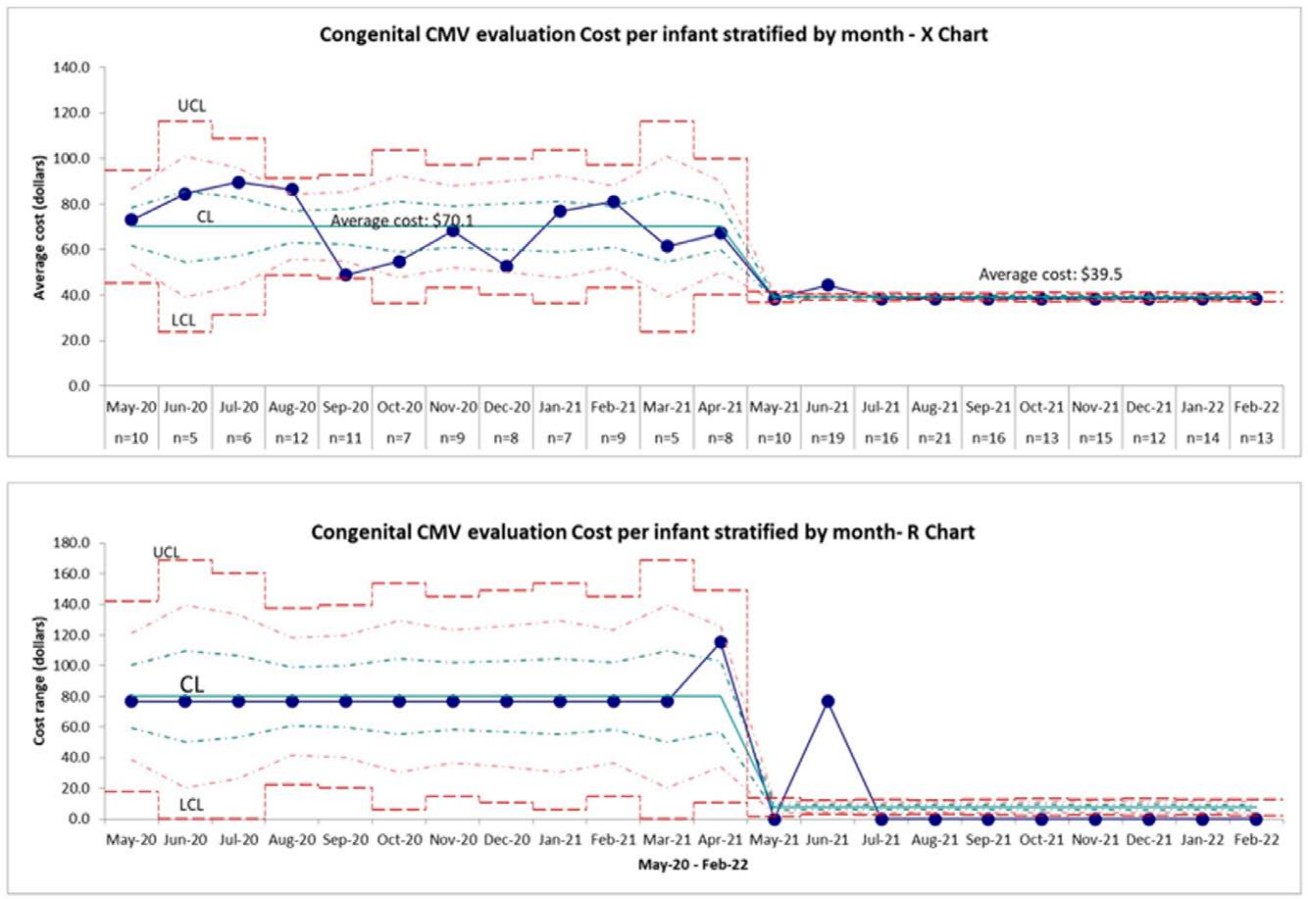
X-bar-R chart depicting congenital CMV infection evaluation cost per infant during each month (outcome measure). The upper chart indicates the monthly averages of CMV evaluation cost per infant and the lower chart depicts the monthly ranges of CMV evaluation cost per infants. The number infants evaluated during each month mentioned along the X axis (n). CMV, cytomegalovirus.

The cost of a CMV DNA PCR test on a single urine specimen was $38.40. As the single specimen collections increased significantly, a notable cost reduction occurred following the implementation of the QI interventions. The statistical process control for the monthly cost of urine CMV evaluation per infant was evaluated by the X-bar-R chart (Fig. 4). A process change occurred after implementing QI interventions, as indicated by the centerline shift, with a decline in monthly average cost per infant from a mean value of 70.1 dollars to a mean value of 39.5 dollars.

None of the screened infants were positive for congenital CMV infection during Epoch 1. However, a total of 6 infants out of 1,796 infants (0.33%) born during the Epoch 2 had urine PCR positive for CMV DNA (*P* = 0.015). As the urine CMV positivity rate was higher during Epoch 2, presumably, the implemented QI intervention did not increase the unintended missed identification of congenital CMV infection in the current study (balancing measure).

## DISCUSSION

We demonstrated a significant reduction in resource utilization which supported the concept of value-based care (ie, quality of health outcomes per dollar spent).^14,15^ By standardizing the practice to 1 specimen at our academic institution, we were able to reduce costs. Previous studies suggest unwarranted practice variation and excessive use of diagnostic modalities are key contributors to health care inefficiency and waste.^16-18^ Going from 3 to 1 specimen has reduced waste on many levels. Laboratory testing, from collection to processing, involves resources including personnel, equipment, and finance. We demonstrated a significant reduction in resource utilization, thereby improving healthcare quality. Shrank et al.^19^ have concluded that potential savings from interventions that reduce waste, ranged from $191 billion to $286 billion in the US healthcare system. They further suggested that implementing effective measures to eliminate waste, as shown in our study, represents an opportunity to reduce the continued increases in US health care expenditures.

Targeted and universal screening strategies can identify neonates with congenital CMV infections. As 80% to 90% of neonates with congenital CMV infections are asymptomatic,^10^ cases of congenital CMV infections can remain unidentified if a targeted CMV screening strategy is not used. Asymptomatic neonates with congenital CMV are still at risk with 10% of them developing late-onset sequelae, primarily sensorineural hearing loss.^10^ The identification of asymptomatic cases could enable the identification of subclinical congenital CMV infection manifestations, for instance: periventricular calcification, which would otherwise necessitate adverse outcome preventive valganciclovir therapy.^20-21^ Furthermore, identifying asymptomatic neonates with congenital CMV could facilitate proper resource utilization for early developmental interventions and parental counseling. Given the merits of universal congenital CMV screening, a highly sensitive and cost-effective screening methodology with a convenient sample collection process is more likely to promote the acceptance of universal screening across diverse neonatal units. This was supported by a recently published study from the National Center on Birth Defects and Developmental Disabilities, Centers for Disease Control, and Prevention (CDC), that estimated that in the United States, several thousand children with congenital CMV infection could potentially benefit each year from newborn CMV screening, early detection, and interventions.^22^ Salivary sample collection is more accessible but carries the risk of false-positive rates because of potential contamination of saliva with CMV in human milk.^23^ Congenital CMV screening with a single urine sample is practical, convenient, and cost-effective. As shown by the current study, the consensus and team-building strategies followed by effective communication have enabled the successful implementation of evidence-based, potentially better standard practice for clinical use at our institution.

Our QI initiative had a few major limitations. First, as risk-based, targeted congenital CMV screening is in practice at our center, not having a guidance algorithm with specified indications for congenital CMV screening to maintain uniformity among providers was a significant limitation. Second, postdischarge follow-up of involved infants was not part of the study. As a result, accurate data on potentially missed cases of congenital CMV following the implementation of single urine sample screening could not be gathered. Third, although outcome data were discussed and plotted periodically, outcome measures plotted over time were not displayed on the unit’s visual display board, which was a lost opportunity to keep practitioners up to date on the project’s progress.

Our study highlighted the efficacy of education, reinforcements, and ongoing constructive discussions with healthcare providers about better practices to reduce healthcare costs and promote the judicious use of laboratory tests when the problem of overuse has been identified. We believe our findings will encourage clinicians to obtain CMV PCR in suspected cases of congenital CMV infections or neonates who failed hearing screening tests. Clinicians could practice a single test with the confidence of not missing an actual case of congenital CMV.

## CONCLUSIONS

We increased the single specimen urine CMV PCR test from 53.6% to 98.6%. We also demonstrated a cost reduction, thus improving resource utilization and reducing medical waste.

## DISCLOSURE

The authors have no financial interest to declare in relation to the content of this article.

## ACKNOWLEDGMENTS

We would like to thank Ms. Nicole Nicksic, MLS ASCP, Reference Laboratory Supervisor for her help with data procurement.

## Supplementary Material


