# Supplementary material for: Successful Implementation of Single Urine Polymerase Chain Reaction Test for Evaluating Suspected Cytomegalovirus Infection in Neonates
Source: Pediatr Qual Saf. 2022 Aug 1;7(4):e586. doi: 10.1097/pq9.0000000000000586 (PMC9345636; doi:10.1097/pq9.0000000000000586)
Supplement: Supplementary file 1 [file pqs-7-e586-s001.pdf]

CMV screening ADC.pdf
228 KB

Eval symCMV-J perinatol.pdf
372 KB

QI-CMV.docx
707 KB

Redbook CMV.pdf
162 KB

Show all 6 attachments (2 MB) Download all Save all to OneDrive - LSU Health Shreveport

I am in the process of developing a team for our QI project on CMV screening. I think a represenation from NNPs and nusery team is very important. I have sent them this information asking to be a part of this project.

**Description:**

I am planning to change our practice of obtaining three CMV urine samples on neonates who are IUGR/SGA at birth. Dr. Pichilingue, our Peds ID faculty, has shown interest in the project.

We can do it as a QI project – I guess we can get away with IRB approval (need to find out the details).

My plan is to obtain last year data on urine CMV from labs record. How many? Any positive? Cost?

Starting July 2021, we should only send Saliva CMV on suspected cases (If positive will discuss with Peds ID).
Collect 6 month prospective data, evaluate the yield and cost.

Aim: Reduce the cost of CMV screening in IUGR/SGA neonates.

A rough draft is attached with some references. Please take a look at advise further.

Once I hear back from the NNPs and nursery team, I will compile the team framework.

**Shabih Manzar,** MD, FAAP, CPHQ
Associate Professor, Department of Pediatrics
Director, Pediatric MSIII Clerkship
Louisiana State University Health Sciences Center
Shreveport, Louisiana

KD [redacted]
*EXTERNAL EMAIL: EVALUATE* I would be interested

MS [redacted]
That's exciting. I am waiting on others to respond. Once I get all the feedback , will compile a team. Thanks Regards, S[redacted]

OO [redacted]
I am interested in your QI. I will read the documents sent in more detail but I am always looking for QI opportunities. [redacted]

MS [redacted]
That's exciting. I am waiting on others to respond. Once I get all the feedback , will compile a team. Thanks Regards, S[redacted]

HY [redacted]
Sounds like a great idea. I would love to participate but I have no idea what my duties will be starting july . if im still staffing nursery would love to participate. Sabeen

MS [redacted]
That's exciting. I am waiting on others to respond. Once I get all the feedback , will compile a team. Tha[redacted]II Pediatri...

**<u>Quality Improvement Project</u>**

**Goal:**

Minimizing routine Urine CMV collection for neonates born with a birth weight of less than 3%.

**Current practice:**

We collect three consecutive Urine samples for CMV PCR testing in neonates born with birth weight (BW) of less than 3% (assuming the neonate is Intrauterine growth restricted (IUGR)).

**Introduction/Background:**

As we know that small for gestational age (SGA) (BW < 3%) could be constitutional, therefore we should differentiate these babies. Second, SGA and IUGR are not synonymous. A neonate born with BW of <3% might not be IUGR (Figure)

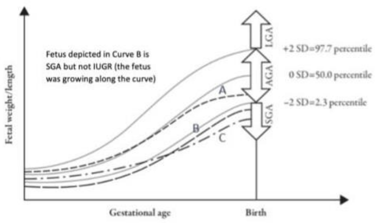


IUGR/SGA could be caused many factors i.e., maternal undernutrition, maternal hypertension, placental insufficiency, twinning (Figure).

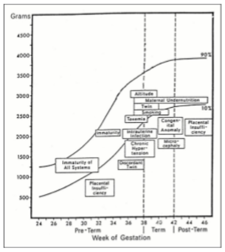


**Congenital Cytomegalovirus (cCMV) infections (why screen):** [1-6]

-screening is simple and possible (saliva /urine-dried blood has low sensitivity)
-most cases are asymptomatic (how likely is that cCMV will present as SGA/IUGR)
-treatment available
-risk of missing cCMV (by not performing the screen)

**QI project (suggestion):**

Collect baseline data:

Yield: We can review the urine CMV PCR data for the last year (look at the details of positive cases)

Intervention: Instead of performing 3 urine CMV tests, we can screen with saliva (good sensitivity and specificity). If the test is positive, we should send the urine (how many samples? should we send three?)

**References:**

1. Hilditch C, Liersch B, Spurrier N, Callander EJ, Cooper C, Keir AK. Does screening for congenital cytomegalovirus at birth improve longer term hearing outcomes? Arch Dis Child. 2018;103:988-992. doi: 10.1136/archdischild-2017-314404
2. Kimberlin DW, Jester PM, Sanchez PJ, et al. Valganciclovir for symptomatic congenital cytomegalovirus disease. N Engl J Med. 2015;372:933-943. doi: 10.1056/NEJMoa1404599
3. Rawlinson WD, Boppana SB, Fowler KB, et al. Congenital cytomegalovirus infection in pregnancy and the neonate: consensus recommendations for prevention, diagnosis, and therapy. Lancet Infect Dis. 2017;17:e177-e188. doi: 10.1016/S1473-3099(17)30143-3
4. Ronchi A, Zeray F, Lee LE, et al. Evaluation of clinically asymptomatic high risk infants with congenital cytomegalovirus infection. J Perinatol. 2020;40:89-96. doi: 10.1038/s41372-019-0501-z
5. American Academy of Pediatrics. Cytomegalovirus Infection. In: Kimberlin DW, Brady MT, Jackson MA, Long SS, eds. Red Book: 2018 Report of the Committee on Infectious Diseases. American Academy of Pediatrics; 2018; 310-317
6. https://www.cdc.gov/cmv/clinical/congenital-cmv.html

Additional references:

https://www.cdc.gov/cmv/clinical/lab-tests.html

https://health.utah.gov/cshcn/pdf/CMV/CMV%20PCR%20Testing.pdf

https://www.findlabtest.com/lab-test/general-wellness/cytomegalovirus-cmv-quantitative-urine-pcr-labcorp-139144
